# A Digital Therapeutic Allowing a Personalized Low-Glycemic Nutrition for the Prophylaxis of Migraine: Real World Data from Two Prospective Studies

**DOI:** 10.3390/nu14142927

**Published:** 2022-07-17

**Authors:** Vivian Valeska Lelleck, Franziska Schulz, Oliver Witt, Gianna Kühn, Dominik Klein, Astrid Gendolla, Stefan Evers, Charly Gaul, Diamant Thaçi, Christian Sina, Torsten Schröder

**Affiliations:** 1 Institute of Nutritional Medicine, University Hospital of Schleswig-Holstein, Campus Lübeck & University of Lübeck, Ratzeburger Allee 160, 23538 Lübeck, Germany; vivian.lelleck@student.uni-luebeck.de (V.V.L.); christian.sina@uksh.de (C.S.); 2 Perfood GmbH, Research & Development, Am Spargelhof 2, 23554 Lübeck, Germany; franziska.schulz@perfood.de (F.S.); oliver.witt@perfood.de (O.W.); gianna.kuehn@perfood.de (G.K.); dominikklein@outlook.de (D.K.); 3 Medical Practice for Neurology and Pain Therapy Essen, Am Alfredusbad 2, 45133 Essen, Germany; a.gendolla@praxis-gendolla.de; 4 Faculty of Medicine, University of Münster, Domagkstr. 3, 48149 Münster, Germany; everss@uni-muenster.de; 5 Department of Neurology, Krankenhaus Lindenbrunn, 31863 Coppenbrügge, Germany; 6 Headache Center Frankfurt, Dalbergstr. 2a, 65929 Frankfurt am Main, Germany; c.gaul@kopfschmerz-frankfurt.de; 7 Institute and Comprehensive Center for Inflammation Medicine, University of Lübeck, Ratzeburger Allee 160, 23538 Lübeck, Germany; diamant.thaci@uksh.de

**Keywords:** migraine prophylaxis, personalized nutrition, continuous glucose measurement, digital therapeutic, low-glycemic diet, low-glycemic index, headache, nutrition, episodic migraine, real world data

## Abstract

Migraine is a headache disorder associated with a high socioeconomic burden. The digital therapeutic sinCephalea provides an individualized low-glycemic diet based on continuous glucose measurement and is intended to provide a non-pharmacological migraine prophylaxis. We performed two prospective studies with migraine patients who used sinCephalea over a period of 16 weeks. The patients used a headache diary and recorded their migraine-related daily life impairments using the assessment tools HIT-6 and MIDAS for a pre versus post comparison. In addition, continuous glucose data of patients were compared to healthy controls. In both studies, patients reported a reduction of headache and migraine days as well as reductions in HIT-6 and MIDAS scores. More specifically, migraine days decreased by 2.40 days (95% CI [−3.37; −1.42]), HIT-6 improved by 3.17 points (95% CI [−4.63; −1.70]) and MIDAS by 13.45 points (95% CI [−22.01; −4.89]). Glucose data suggest that migraine patients have slightly increased mean glucose values compared to healthy controls, but drop into a glucose range that is below one’s individual standard range before a migraine attack. In conclusion, sinCephalea is a non-pharmacological, digital migraine prophylaxis that induces a therapeutic effect within the range of pharmacological interventions.

## 1. Introduction

Migraine is currently the third most common disorder worldwide [1]. The one-year prevalence ranges from 10 to 15% [2,3,4]. Migraine is a severe neurological disorder in which persistent headaches are prominent and force patients to withdraw from sensory influences (e.g., light, noise, movement). These migraine attacks are often accompanied by other symptoms such as nausea, vomiting or even temporary neurological symptoms such as paralysis or visual disturbance [5]. At the same time, more than half of migrane patients report that their performance and daily life are affected by their headaches, and up to 25% of migraine sufferers report a loss of productivity due to absenteeism and presenteeism at work [6,7]. The direct healthcare costs for migraine in Germany are estimated to be 450 million Euro per year [8], while the indirect health care costs are expected to be up to 7 times higher [9]. In the United States, the direct and indirect costs amount to approximately 36 billion dollars [10]. These data indicate that migraine is not only a common and severely debilitating condition, but also associated with a high level of economic impact.

Migraines are usually treated with medication for attack therapy, and if necessary, for attack prophylaxis. Analgesics and triptans are mainly used for acute treatment of migraine attacks. However, regular use of analgesics carries the risk of drug-dependent headaches and other drug side effects [11]. Because of these risks and the interference with daily life caused by migraine attacks, the current guideline recommends pharmacological prophylaxis when a person experiences three migraine attacks per month [12]. This is usually done with drugs that were originally intended for other indications, such as beta-blockers, calcium blockers, antiepileptics or antidepressants. These drugs are used because a migraine attack-reducing effect has been observed as a side effect with these drugs [13]. However, drug approaches have numerous additional side effects such as dizziness, diarrhea, fatigue, weight gain and erectile dysfunction [12]. These side effects are probably the main reason why drug compliance is very low [14,15]. A new, and so far only, specific migraine pharmacological strategy is antibodies against the neuropeptide Calcitonin Gene-Related Peptide (CGRP) or its receptor [16]. However, currently this approach is reserved for special cases and not routinely offered to all patients [17,18].

An adjuvant non-pharmacological therapy and prophylaxis is recommended by many experts [12]. These include stress-reducing measures such as muscle relaxation or biofeedback, regular moderate endurance sports, increased hydration, fixed daily rhythms, consistent patterns of sleep and shorter intervals between eating.

Several studies have found a close association between insulin resistance as well as associated elevated blood glucose, and insulin levels and migraine [19,20,21,22]. This suggests that a central nervous energy deficiency due to insufficient energy supply by glucose plays a role in the development of migraine attacks. The brain’s glucose energy supply is regulated by the neuropeptides CGRP and Pituitary Adenylate Cyclase-Activating Polypeptide (PACAP), which are also considered key peptides for the pathophysiology of migraine in current migraine research [23,24]. Several PACAP and CGRP inhibitors are currently in development or in clinical trials. Effective regulation of glucose homeostasis in the brain is therefore a promising strategy for effective migraine prophylaxis.

Diets that stabilize blood glucose levels are reported to improve migraine symptoms. A three-month carbohydrate-modified diet reduced headache days and pain duration and intensity in a study of 50 migraineurs [25]. Another twelve-week low-glycemic diet intervention with 350 participants showed a significant reduction in pain intensity and frequency of seizures in migraine patients. The authors concluded that a low-glycemic diet is an effective and reliable method of migraine prophylaxis without risks of adverse drug effects [26]. This is further supported by an analysis conducted as part of the medical device development of sinCephalea, which showed clear signs of efficacy of a personalized low-glycemic approach. In addition, the analysis revealed an increasing interest among migraine patients in a specific nutritional therapy as an alternative to drug therapies with unfavorable side effect profiles [27]. The digital therapeutic (DTx) sinCephalea is designed to take into account that postprandial blood glucose metabolism is regulated differently inter-individually, and that dietary recommendations to ensure sustained low blood glucose levels must be personalized based on individual blood glucose metabolism [27,28,29,30,31]. sinCephalea uses the concept of a personalized low-glycemic diet and enables applicability through digital personalization. The personalization of dietary recommendations leads to significantly higher treatment adherence [32].

We conducted two prospective studies with migraine patients who used sinCephalea over the course of 16 weeks. Essential components of this program are a continuous glucose testing phase, in which individual blood glucose responses to meals and test meals are analyzed, and the provision of a personalized nutrition report enabling the implementation of a low-glycemic diet. The first study aimed at proving the applicability of sinCephalea and showing first indications of clinical effectivity. The second aimed at independently recapitulating these findings and to evaluate clinical effectiveness.

## 2. Materials and Methods

### 2.1. SinCephalea

The DTx sinCephalea is a medical device under the European Union Medical Device Regulations (MDR). sinCephalea creates personalized nutritional recommendations for a low-glycemic diet. The starting point for the development of sinCephealea was the finding from our own research lab that migraine patients reported noteworthy clinical improvements after receiving low-glycemic diet recommendations outside of a clinical study setting [27].

The sinCephalea dietary recommendations are based on continuously measured tissue glucose levels. For this, the patients continue their usual dietary habits and wear a device for continuous glucose monitoring (CGM) for at least ten days. In addition to their usual diet, the patients can consume standardized test meals that are suitable for comparing interchangeable foods (e.g., white bread instead of wholegrain bread for breakfast or potatoes instead of pasta or rice for lunch). These foods correspond to foods that are typically eaten frequently and are alternatives to each other. The aim of the test meals is to provide patients with easy-to-follow personalized recommendations.

All CGM data are collected and processed by the hardware and software of the respective manufacturers. Afterwards, the data is then imported into sinCephalea for further processing and analysis. Following these steps, each patient receives a personalized report for a low-glycemic diet, which forms the basis of the migraine prophylaxis. In the report, all logged meals are ranked according to their individual glycemic effect, and thus, on energy supply to the brain. In addition, the analysis of the test meals allows for providing simple nutritional rules and enables patients to adjust their eating habits in order to assure a stable postprandial glucose pattern. Furthermore, the report presents a detailed evaluation of the diet during the test phase based on macronutrients, calories and dietary fiber, as well as information about the usual guidelines for a healthy and balanced diet, e.g., the recommendation of the German Nutrition Society [33]. Of note, this approach is not a restrictive diet, which excludes all carbohydrate-containing foods. Neither is it a so-called “migraine diet”, e.g., based on eliminating food components reported to trigger migraine attacks [34,35]. Personalized food recommendations means that only the specific foods responsible for undesirable high-glycemic reactions in the respective individual, are targeted for reduction.

Parallel to food intake, the digital therapeutic app records events such as migraine attacks as well as their symptoms via a headache diary. The personalized nutritional recommendations are supplemented with educational lessons on migraine and nutrition as well as other lifestyle rules for migraine, such as stress reduction and sleep hygiene. This allows patients to understand their disease and the nutritional therapy. It also helps patients to adapt to the changes more easily and to stick to them [36,37].

In summary, sinCephalea is intended to add an option to prevent migraine attacks using a non-pharmacological approach based on personalized low-glycemic nutrition. Over a twelve-week intervention phase, patients are gradually supported to implement their personalized nutrition recommendations into their daily lifestyle.

### 2.2. Study Design: Real World Data Analyses

We performed two independent prospective intervention studies obtaining real world data [38,39]. The first was mainly focused on collecting initial patient-centric data using the sinCephalea DTx as migraine prophylaxis and to assess the applicability of this novel DTx. The second study was designed to assess clinical data more accurately in order to evaluate for clinical effectiveness. However, both studies followed the same design.

**Figure 1:**
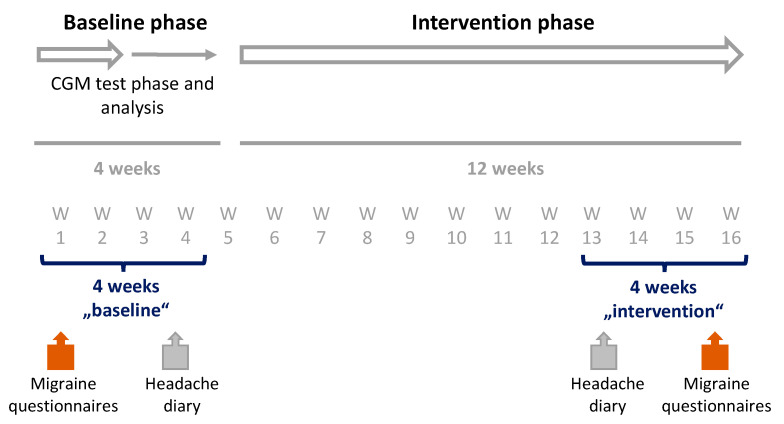
Study design. Patients used the DTx sinCephalea over the course of 16 weeks. The baseline phase was set to be the first four weeks in which patient’s glucose reaction were recorded with a continuous glucose monitoring device (CGM) and the personalized dietary recommendations were determined. Disease severity was assessed with a headache diary and validated migraines questionnaires on impairment in daily life (Headache Impact Test 6-items [HIT-6] and Migraine Disability Score [MIDAS]), and quality of life (EQ-5D-5L). This was followed by a twelve-week intervention phase, in which dietary recommendations were implemented. In the last four-weeks of intervention phase, the disease severity was assessed again for an intra-individual pre-post comparison.

Eligible patients received free access to sinCephalea. Patients used the DTx over the course of 16 weeks as described above. The baseline phase was set to be the first four weeks in which the patient’s glucose reactions to their normal diet and test meals were recorded for evaluation, before receiving the dietary recommendations. The baseline phase was also conducted to prospectively assess disease severity. Disease severity was defined by the number of migraine headache days in a 28-day period. The baseline phase was then followed by a twelve-week intervention phase. During this phase, patients were instructed to follow their personalized nutrition and lifestyle recommendations (Figure 1). The first access code of the first study was activated on 30 September 2020 (“first patient in”). Data collection ended on 21 April 2021 (“last patient out”). The first access code of the second study was activated on 19 November 2021 (“first patient in”). Data collection ended on 6 May 2022 (“last patient out”). The study design follows the recommendations of the International Headache Society for conducting trials with medications for the prophylaxis of migraine as well as the recommendations for the use of health technologies in the treatment of migraine [40,41].

### 2.3. Assessment Tools

Headache data was collected prospectively by documenting all migraine attacks via an electronic headache diary. The two observational studies only differed in the procedure of tracking the days with headache. In the first study, patients recorded headaches on demand, i.e., when the headache attacks occurred. In the second study, all patients used a headache diary on a daily basis. Additionally, symptom-free days had to be actively recorded in the second study. Furthermore, the headache diary in the second study was designed to distinguish between days with typical migraine symptoms according to the ICHD-3 criteria [42] (qualifying these days as migraine days) from non-migraine days for headaches not qualifying as migraine attacks. The electronic headache diary was designed according to the recommendation of the International Headache Society [40,41]. For the evaluation of changes of the disease severity, headache or migraine days of the first four weeks (“baseline”) were compared with those of the last four weeks of the intervention phase (“intervention”).

At the beginning of the baseline phase and at the end of the intervention phase, the patients answered validated questionnaires on impairment in daily life, namely Headache Impact Test-6 items (HIT-6), Migraine Disability Score (MIDAS) and quality of life (EQ-5D-5L), within the sinCephalea app. The use of these questionnaires is recommended by the International Headache Society for the implementation of studies with drugs for prophylaxis in patients with migraine [41].

The HIT-6 questionnaire describes migraine-related impairment in everyday life [43,44]. A five-point scale is used to indicate how often everyday activities were impaired [45,46]. The level of the score (from 37 to 78) reflects the impairment. The score is tabulated by adding the six items. Each item can be answered with “never” (6 points), “rarely” (8 points), “occasionally” (10 points), “very often” (11 points) or “always” (13 points). The HIT-6 covers a period of four weeks.

The MIDAS questionnaire measures headache-related disability [47,48,49]. The MIDAS is validated for use over a twelve-week period. Patients are asked to rate all headache attacks in the last twelve weeks. They are asked to indicate the total number of days in this period where they were unable to attend work or school (absenteeism) or only able to do so to a limited extent (presenteeism), on which they were unable to do housework or only able to do so to a limited extent, and how often they were unable to take part in leisure activities. The MIDAS score indicates the headache-related impairment as the sum of the days mentioned. The score obtained was compared at baseline (refers to the twelve weeks before the start of the application) and at the end of the intervention phase, which refers to the twelve weeks of the intervention phase.

The EQ-5D-5L questionnaire captures health status and quality of life [50]. Its use is recommended for migraine studies [41,51,52]. It asks about five dimensions/life domains: pain, mobility, self-care, activities of daily living and anxiety. An index value can be calculated from the answers, which is created on a country-specific basis using a reference cohort and summarizes all dimensions [53]. To measure the changes in quality of life, the index value was calculated according to the licensors’ specifications and compared at the beginning of the baseline phase and at the end of the intervention phase.

The patients’ general impressions of the change in their general condition were recorded once at the end of the intervention phase using the PGIC (Patient Global Impression of Change). They indicated whether their general condition had become “very much better” (1), “much better” (2), “a little better” (3), “unchanged” (4), “a little worse” (5), “much worse” (6) or “very much worse” (7) since the beginning of the study.

In the intervention phase, adherence to personalized dietary recommendations was assessed weekly by means of a questionnaire in the app.

### 2.4. Study Population

In both studies, data of patients with between 18 and 65 years of age with episodic migraine according to the International Classification of Headache Disorders (ICHD-3) for migraine without aura (diagnosis 1.1 of ICHD-3) and migraine with aura (diagnosis 1.2 of ICHD-3, all subtypes) with at least three migraine headache days per month, no evidence of chronic migraine in the last three months, onset of migraine before 50 years of age, and presence of the disorder for at least twelve months were analyzed. In the first study, migraine day frequency was assessed at inclusion, and all data from all patients were analyzed irrespective of migraine frequency in the baseline phase. In the second study, a minimal migraine day frequency of three migraine days per month was additionally verified in the baseline phase, and only data from patients with at least three migraine days in the four-week baseline phase were analyzed.

In addition, sufficient knowledge of German was required to understand the study documents. To use the sinCephalea app, a smartphone with Android (version 5.0 or higher) or iOS (version 12.0 or higher) was required. Patients with insulin-treated diabetes mellitus were excluded from participation. Subjects who participated in another study at the same time or who had previously used sinCephalea were not included.

### 2.5. Glucose Data Analysis

In addition to the clinical efficacy data, we analyzed dietary and glucose data from the CGM test phase of 49 migraine patients and compared this data with data from 103 healthy individuals. This “healthy reference cohort” was an age-, BMI-, and sex-matched subset of a total of 1059 individuals, who reported to be healthy. The dietary and glucose data was collected as part of a digital nutrition program of Perfood GmbH, in which the participants voluntarily consented to anonymous data analysis. This data is not yet published; however, the digital nutrition program is described elsewhere [27].

For the comparison of the dietary data, mean daily intake of calories, macronutrients, and fiber were analyzed. In addition, meal frequency and the mean length of the overnight fasting period were considered. Glucose data was compared by mean time in the glucose ranges below 80 mg/dL (4.4 mmol/L), between 80 and 130 mg/dL (4.44–7.22 mmol/L), and above 130 mg/dL (7.22 mmol/L).

A separate analysis was performed with data from the migraine patients only. For this, we intra-individually compared CGM characteristics in 24-h windows before the onset of migraine symptoms to 24-h windows not preceding migraine symptoms (of each respective person).

### 2.6. Statistical Analysis

The primary clinical endpoints of these studies are the intra-individual change in the number of days with headaches (first study) or migraine headache per month (second study) in the last four weeks of the intervention phase compared to the baseline phase. The days with headaches were recorded using the electronic headache diary. In the first study, the headache diary was filled out on demand, while daily recording was mandatory in the second study. The headache diary used in the second study was designed according to the recommendation of the International Headache Society [40,41]. Based on the questions in the headache diary, it was possible to distinguish between headache and migraine-type headache.

In the first study, data from the headache diary was analyzed as reported. For the second study, the following strict rules applied: If patients had provided information in the headache diary for at least 22 days (80%) per 28 day-period but less than 28 days, the missing data was prorated. The missing number of days was replaced proportionally by the average of valid entries per 28 days. If fewer than 22 days were recorded in the headache diary during the intervention phase, the days were imputed with individual migraine frequency data from the baseline. This ensured that the actual headache diary data was considered and only the missing data was replaced.

In both studies, the data from the other questionnaires were evaluated if data was available at both measurement times.

For all analyses, a paired, two-sided *t*-test (significance level 5%) was applied to test the means at baseline and at intervention. In the absence of normal distribution for samples or ordinal scale level, the Wilcoxon test was performed.

The following applied for the second study: Patients who reported at least 22 days (80%) of headache diary entries and three or more migraine headache days during the baseline phase were included in the systematic data analysis. Dropouts and individuals who reported less than 22 days of data during the last four weeks of the intervention phase were included using imputation with baseline-observation-carried-forward (BOCF) to allow an intention-to-treat (ITT) analysis.

### 2.7. Adverse Events

Adverse events were reported and documented in accordance with ISO 14155:2020. Headaches were not documented as adverse events since they were already documented in the headache diary and analyzed as clinical endpoints.

In the first study, a total of four patients (8%) reported adverse events of which one was classified as serious adverse events (tooth surgery *n* = 1). The other adverse events involved an infection with COVID-19 (*n* = 1), a calcaneal spur (*n* = 1) and dental problems (*n* = 1). No device deficiencies were recorded.

In the first study, a total of 16 patients (23%) reported adverse events of which two were classified as serious adverse events (arm surgery *n* = 1, nose surgery *n* = 1). The most common adverse event in patients was an infection with COVID-19 (*n* = 10). The other adverse events involved a disc prolapse (*n* = 1), sudden hearing loss (*n* = 1), ulcerative colitis flare (*n* = 1) and the occurrence of a depression (*n* = 1). The occurrence of depression led to the discontinuation of the study. No device deficiencies were recorded.

## 3. Results

### 3.1. First Study Collecting Real World Data from Patients Using sinCephealea as Migraine Prophylaxis

#### 3.1.1. Baseline Demographics of First Study Population

Real world data from 49 patients with episodic migraine using sinCephalea over the course of 16 weeks were analyzed. At baseline, the cohort had a mean age of 41 years (standard deviation (SD) 9.19), a body mass index (BMI) of 27.1 kg/m^2^ (SD 7.31), 87.8% were female (*n* = 43). The mean duration since migraine diagnosis was 24.6 years (SD 19.42). 34.7% were taking prophylactic medications; most commonly magnesium (16%), beta-blockers (4%), topiramate (4%) or amitriptyline (2%). Acute medications were used regularly by 98%, with ibuprofen (51%) and triptans (49%) reported most frequently. Headaches were reported to be usually accompanied by migraine-typical symptoms such as photophobia/phonophobia, nausea or vomiting in 98%, and 69.4% had migraine with aura.

#### 3.1.2. Baseline Disease Severity

At baseline, the 49 patients had 4.26 headache days (SD 3.36) with a mean intensity of 6.29 (SD 1.37). Impairment assessed with HIT-6 was 61.80 points (SD 2.60) and assessed with MIDAS 37.6 points (SD 58.9). Quality of life was assessed using EQ-5D-5L. The index value was 0.891 (SD 0.11) (Table 1).

#### 3.1.3. Comparison of Disease Severity between Baseline and End of the Intervention Phase

We compared the disease severity at the end of the twelve week intervention phase (“intervention”) with the baseline disease severity (Table 1). The number of headache days per four weeks decreased by a mean of 2.43 days (SD 3.19, *p* < 0.001, 95% CI [−3.36; −1.49]) to a mean headache day frequency of 1.83 headache days per month (SD 3.50). 73.5% of all patients reported at least a 50% reduction in migraine days (50%-responder rate).

In addition, the HIT-6 score decreased by 4.48 points (SD 5.67, *p* < 0.001, 95% CI [6.82; 2.60]) and the MIDAS score decreased by 11.03 points (SD 28.81, *p* < 0.01, 95% CI [−21.87; −0.44]). The relative improvement in the EQ-5D-5L index value was 4.5% (SD 10.84, *p* < 0.05, 95% CI [−0.06%; 7.89%]) as the index value increased to 0.942 (SD 0.08).

In addition, we performed a follow-up analysis approximately one year (325–355 days) after study participants received their personalized nutrition report. 15 patients reported the number of headache days in the preceding three-month period as well as the current HIT-6 and MIDAS scores. Compared to the initial baseline phase, the number of headache days in the last three months before the survey decreased by 7.67 days (SD 5.89, *p* < 0.001, 95% CI [−10.93; −4,4]). In addition, HIT-6 decreased by 2.94 points (SD 3.43, *p* < 0.001, 95% CI [−4.17; −1.7]) and MIDAS decreased by 26.6 points (SD 52.92, *p* < 0.001, 95% CI [−55.9; −2.70]). No data from the patients not taking part in the follow-up survey could be analyzed.

In summary, the analysis of headache days and additional patient-centric metrics showed a significant reduction after the use of the DTx sinCephalea for 16 weeks as well as after almost a year from receiving the personalized nutrition recommendations.

#### 3.1.4. Continuous Glucose Measurement

**Figure 2:**
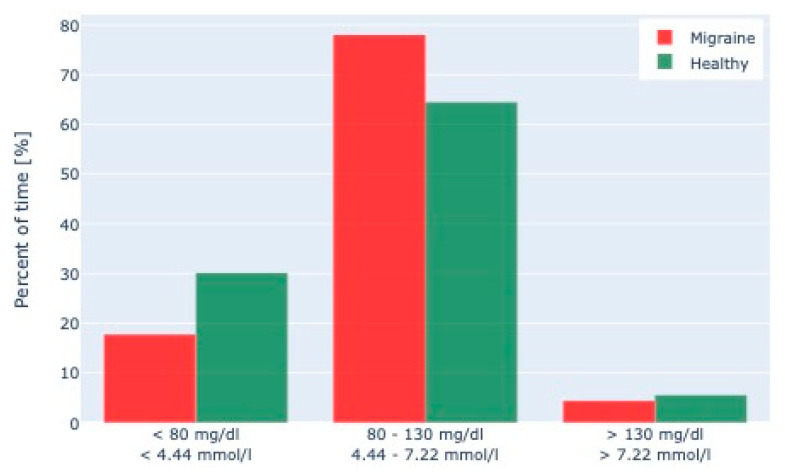
Continuous Glucose Monitoring (CGM) data of migraine patients and healthy patients. A comparison of CGM data of 49 migraine patients with those of 103 healthy individuals matched for BMI, age, and weight showed that migraine patients spent significantly more time in a glucose range between 80–130 mg/dL and less in the range < 80 mg/dL.

The comparison of dietary and glucose data from the first study to a healthy control cohort revealed no significant difference in macronutrients (carbohydrates, *p* = 0.42; protein, *p* = 0.57; fat, *p* = 0.77), energy (*p* = 0.53) and fiber intake (*p* = 0.53), as well as average duration of overnight fasting (*p* = 0.35) and meal frequency (average number of meals per day and person, *p* = 0.87). However, migraine patients spent significantly more time in the glucose range from 80 to 130 mg/dL (39.7%, *p* = 0.003) and less in the range below 80 mg/dL (72.2%, *p* < 0.001) on average over the entire CGM test phase (Figure 2). This increase in mean glucose levels appeared more pronounced at night than during the day (mean glucose at night 6.0% higher, *p* < 0.001, mean glucose during the day 3.9% higher, *p* = 0.018).

In addition, an intra-individual comparison of the 24 h preceding a migraine attack (a total of 193 migraine attacks in the 49 migraine patients) with the mean of the 24-h periods without a following attack (for each respective person) showed that migraine patients drop significantly longer into a blood glucose range one standard deviation below their CGM mean before an attack. In particular, the time spent in this relatively and individually low glucose range increased by 26.2% (*p* = 0.032) in the 24-h periods before an attack compared to the 24-h periods with no following attack.

Taken together, these analyses of CGM and headache data of migraine patients indicate that migraine patients have slightly elevated mean glucose levels but drop below their individual normal prior to a headache attack, pointing towards an individually unstable glucose control before a headache attack.

### 3.2. Recapitulation in a Second Study Collecting Real World Data from Patients Using sinCephealea DTx as Migraine Prophylaxis

The first clinical study prospectively collecting data clearly suggested that sinCephalea could act as a prophylactic treatment in patients with episodic migraine. However, the data was collected with an electronic diary that only allowed enumerating the reported headaches, and no adjustments for non-reported headaches could be made. To recapitulate the findings and to assess more tightly controlled data, we performed a second one-armed study with patients with episodic migraine. Of note, all these patients used sinCephalea for the first time.

#### 3.2.1. Patient Flow

A total of 97 patients activated the access code to the DTx. Four patients discontinued use prematurely (reasons given: participation not compatible with time *n* = 1; participation no longer possible due to other illness [depression] *n* = 1; no further interest due to new prophylactic medication *n* = 1; lost to follow-up *n* = 1).

71 patients provided information in the headache diary on at least 22 days of the baseline phase (“baseline”) and reported migraine headaches on three or more days. Based on the regular entries in the headache diary, their disease severity at baseline could be reliably be determined. 87% (*n* = 62) also regularly completed the headache diary during the last four weeks of the intervention phase (“intervention”) and could be included in the complete data set analysis. The data from the other nine patients were imputed using BOCF for the ITT analysis.

**Figure 3:**
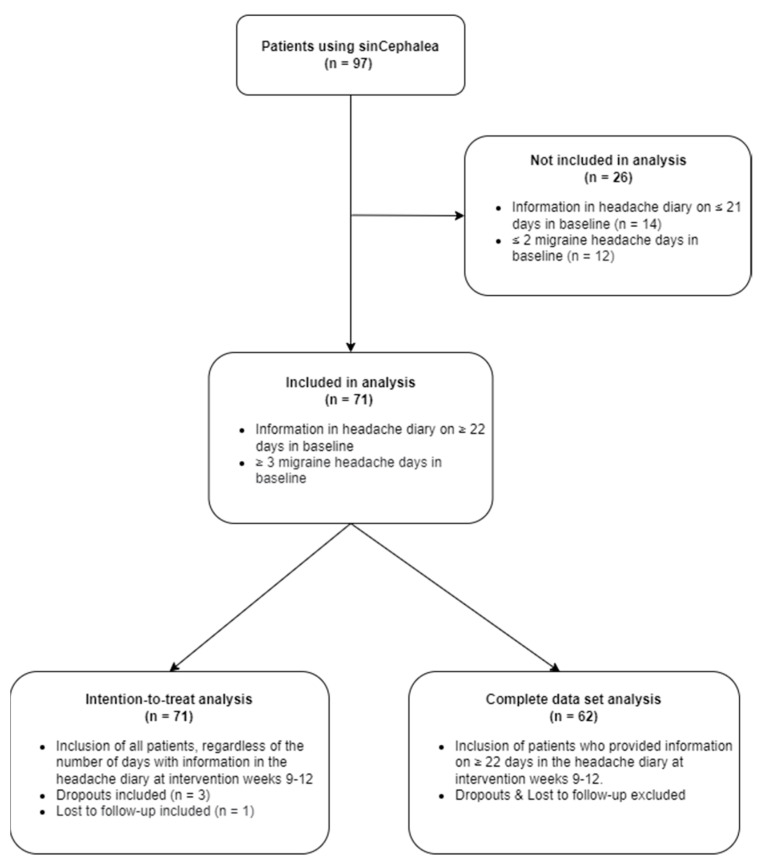
Flow chart of patients included in the second study. 97 patients activated the access code to the DTx sinCephalea. 71 patients were included in the analysis as they fulfilled the criteria of completed headache diary on at least 22 days and reported migraine headache on at least three days in the baseline phase. 62 patients also provided information on at least 22 days of the last four weeks of the intervention phase and were included in the completed data set analysis. Missing data of the last four weeks of the intervention phase from the remaining nine patients was analyzed using imputation with baseline-observation-carried-forward (intention-to-treat analysis).

Endpoints from the migraine questionnaires were available from *n* = 60 patients at both survey time points. The PGIC was answered by a total of 64 patients (Figure 3).

#### 3.2.2. Baseline Demographics of Second Study Population

A total of 71 patients could be included in the analysis of the second study. They all reported at least twenty-two days of baseline in the headache diary, and reported migraine headaches on three or more days. At baseline, the 71 patients were on average 40 years old (SD 12.33), had a mean BMI of 24.75 kg/m^2^ (SD 5.48) and were 94% female (*n* = 67). The mean time the patients already had the migraine diagnosis was 22.51 (SD 12.22) years. 54% (*n* = 38) were taking prophylactic substances. The most common prophylactic substances were magnesium (28.07%) and combined preparations with magnesium plus vitamin B2 plus coenzyme Q10 (14.04%) as well as antidepressants (21.13%), CGRP antibodies (17.54%), and beta-blockers (8.77%). Patients were instructed to keep their prophylactic medication unchanged for the duration of the data collection. Patients reported taking an acute medication (pain relievers such as ibuprofen or triptans) on a mean of 6.42 (SD 2.55) days per month.

#### 3.2.3. Baseline Disease Severity

At baseline, the 71 patients had 10.33 (SD 4.09) headache days. Of these, 7.78 (SD 3.92) could be classified as migraine headache days (Table 2). On 2.55 (SD 2.98) days, headaches were present that did not fulfil the criteria of a migraine headache. At inclusion, patients confirmed that they had had fewer than fifteen days of headache per month in the last three months; thus, we excluded patients with chronic migraine from this analysis.

Impairment assessed with HIT-6 was 64.43 points (SD 3.85) and assessed with MIDAS 53.57 points (48.28). Quality of life was assessed using EQ-5D-5L. The index value was 0.87 (SD 0.17).

#### 3.2.4. Reduction of Migraine Headache from Baseline to Intervention

The number of migraine headache days per month decreased significantly in the intervention in an intra-individual comparison to baseline (difference: −2.40 days, *n* = 62, *p* < 0.001, 95% CI [−3.37; −1.42]). This corresponded to a median 44% reduction. A significant reduction was confirmed when missing values were imputed (BOCF) in the ITT analysis (difference: −2.23 days, *n* = 71, *p* < 0.001, 95% CI [−3.10; −1.37]) (Table 2).

58% (*n* = 36) of patients could be classified as 30%-responders and 47% (*n* = 29) as 50%-responders. In the ITT dataset, 53% met the 30%-responder status (*n* = 38) and 42% met the 50%-responder status (*n* = 30).

**Figure 4:**
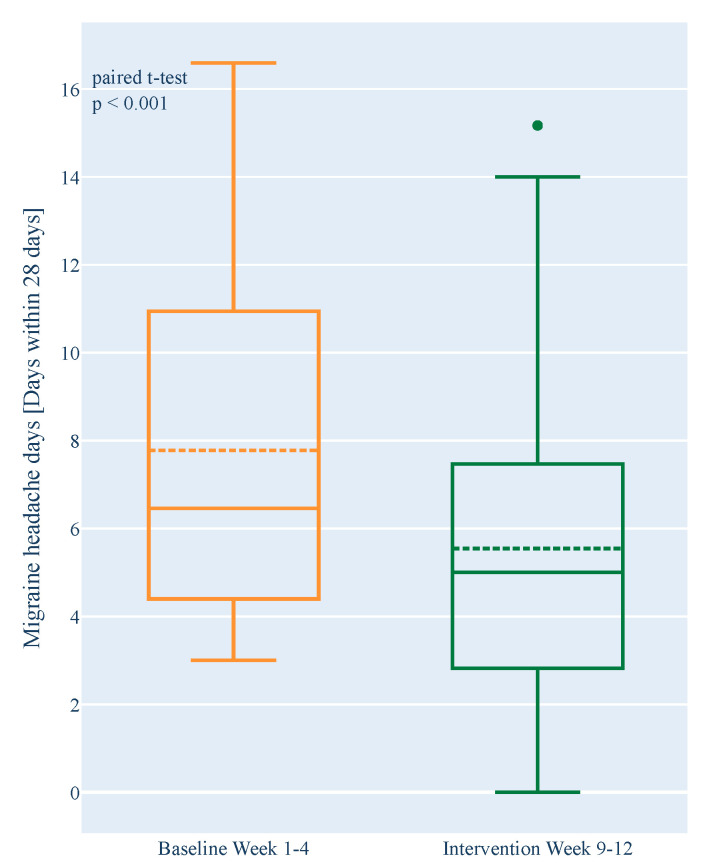
Change in the number of monthly migraine headache days between the four-week baseline phase and the last four weeks of the intervention phase in the complete data set analysis (*n* = 62). Number of headache days was assessed by daily headache diary. Boxplots show 1st quartile, median (solid line), mean (dashed line), and 3rd quartile. Outliers are marked with dots.

The number of non-migraine headache days also decreased significantly (difference: −1.26 days, *n* = 62, *p* < 0.001, 95% CI [−1.79; −0.72]) (Figure 4). A significant reduction was confirmed when missing values in the IIT analysis were imputed (BOCF) (difference: −1.14 days, *n* = 71, *p* < 0.001, 95% CI [−1.62; −0.66]).

The cumulative duration of migraine headaches in hours per month decreased significantly compared to baseline (difference: −15.22 h, *n* = 62, *p* = 0.005, 95% CI [−25.54; −4.90]).

#### 3.2.5. Migraine Questionnaires

Migraine-related impairment in daily life, measured by the HIT-6 questionnaire, decreased significantly at intervention compared to baseline (difference: −3.17, *n* = 60, *p* < 0.001, 95% CI [−4.63; −1.70]). Headache-related impairment (MIDAS) decreased significantly over the three-month intervention period compared with the three months before study participation (difference: −13.45, *n* = 60, *p* = 0.002, 95% CI [−22.01; −4.89]. Quality of life (EQ-5D-5L) showed a slight, statistically not significant improvement (difference: +0.022, *n* = 60, *p* = 0.240, 95% CI [−0.03; 0.08]) (Table 3). 80% (*n* = 51) of the patients reported a subjective improvement in their general condition in the PGIC (score 1, 2, 3).

## 4. Discussion

We present real world data from two independent prospective studies, in which patients with episodic migraine used the DTx sinCephalea for the prophylaxis of their migraine. In both studies, patients reported a significant reduction in the number of headache and migraine days, accompanied by improvement in quality of life and decrease in migraine-related impairment of daily life.

The implementation of such an innovative DTx for the reduction of migraine day frequency meets an important medical need. Migraine experts and professional societies increasingly recommend adaptation of the diet for migraine prophylaxis. For example, the National Headache Foundation [54], the American Migraine Foundation [55,56] and the German Migraine and Headache Society [57] published dietary recommendations for migraine patients. The importance of nutritional adjustments is the subject of various clinical studies and review papers on which these recommendations are based [25,26,27,35,58,59,60,61,62,63,64,65].

In addition, it is reported that the medical care situation of migraine patients is insufficient. More than half of migraine patients do not receive frequent medical care [6] and one-third of patients are not treated in accordance with clinical guidelines [66]. Further research suggests that 80% of patients with episodic migraine discontinue prophylactic medication within the first year [14,15]. This significantly increases the risk of medication overuse, headaches and secondary complications from the medication use [67,68,69].

Low-glycemic diets have been shown to improve migraine symptoms [25,26,27]. It has been demonstrated repeatedly that postprandial blood glucose metabolism is regulated differently between individuals, and that dietary recommendations to ensure sustained low blood glucose levels should be personalized based on individual blood glucose metabolism [28,29,30,31]. The DTx sinCephalea was developed to facilitate personalized low-glycemic dietary recommendations based on individual metabolic glucose responses, and to provide these recommendations in a structured, digital intervention for migraine patients.

Both study cohorts consisted of patients with migraine according to ICHD-3. The patients in the cohorts represent a suitable cohort to evaluate the clinical effectiveness of the DTx sinCephalea. Parameters such as age, BMI, gender distribution, duration of migraine diagnosis and use of migraine medication were within the expected range of typical clinical studies with migraine patients [70,71,72,73,74,75]. With a baseline migraine severity of 4.26 (SD 3.36) headache days in the first study, these patients were less severely affected than in the second study. The patients within second study reported at baseline 7.78 (SD 3.92) migraine days, and can therefore be considered representative of the group of migraine patients for whom prophylactic therapy is recommended according to guideline recommendations [12], and to whom sinCephalea is accordingly aimed.

Both studies followed the same scheme. The only difference was the assessment of headaches. The headache diary in the first study assessed the number of headache days. The headache diary in the second study allowed distinguishing between migraine and non-migraine headache days, as well as between days with no (migraine) headache and days with missing data input.

The data from the first study clearly indicated clinical effectiveness, as it revealed a mean reduction of headache days of 2.43 (SD 3.19; *p* < 0.001), which corresponds to an average reduction of 62.5%. In addition, during the intervention, median individual improvement in MIDAS score was 45.5% (reduction of 11 points), HIT-6 was reduced by 13% (*p* < 0.001, before 62.0, after 57.5 points) and quality of life increased by 4.5% (SD 10.84; increase from 0.891 to 0.942; *p* < 0.05). Moreover, a small proportion of the participating patients still reported improvement in these parameters one year after.

These strong data provide a first indication of the potential use of sinCephalea in migraine therapy. The relationship between a low-glycemic diet and the reduction in migraine headache days may be induced by the stabilization of glucose excursions after meal intake. In fact, the CGM data analysis revealed slight but consistent overall increases in glucose values compared to healthy controls, and drops into ranges which are below what is normal for each individual before migraine attacks. These preliminary findings demonstrate a potential aberrant glucose metabolism in migraine patients, and are in line with reports about increased insulin levels in migraine patients [19,76]. In addition, these findings present a possible functional explanation of why a low-glycemic intervention exerts clinical beneficial effects. Of interest, it has been shown that CGRP secretion is closely linked to glucose metabolism [77]. CGRP act on glucose values and can induce relative hyperglycemia [78]. CGRP levels are also elevated in patients during a migraine attack [79], which is discussed to be as a consequence of a potential migraine-specific central nervous energy deficit due to excessive energy expenditure before the attack [80]. However, it is uncertain whether a hyperinsulinemia observed in migraine patients represents a counterregulatory response to CGRP elevation [19,76] or might even be the root cause.

The aim of sinCephalea is to provide a non-pharmacological option to effectively reduce migraine days as a supplement to the current standard of care. To recapitulate the findings of the first study, we performed a second, independent study. It was intended to demonstrate that the use of sinCephalea provides a medical benefit for patients with migraine. The number of monthly migraine headache days decreased significantly by 2.40 days (95% CI [−3.37; −1.42]) compared to baseline. After imputation of missing values in the ITT data set, the reduction was still 2.23 days (95% CI [−3.10; −1.37]). In parallel, the cumulative duration of migraine headaches decreased by 15.22 h. Migraine-related impairment in daily life, assessed by the HIT-6 questionnaire, decreased by 3.17 points. The MIDAS also decreased by 13.45 points during the intervention period. Moreover, patients reported a high rate of usage of the app-based digital therapeutic. 89% of study patients adhered to their personalized dietary recommendations. They adjusted half or more of all meals eaten to fit their individual recommendations (mean meal adherence was 72.5%, data not shown). 62 of 71 patients (87%) used their headache diary daily and at the end of the intervention 80% of the patients indicated that they felt their migraine had improved during the course of using sinCephalea. Thus, it can be concluded that sinCephalea results in both high patient engagement and subjective therapeutic effectiveness for the majority of patients.

To systematically classify the reported clinical effects, we compared our data with thresholds for minimal clinically important differences. A threshold of 30% reduction in migraine days is reported for non-pharmacological interventions [81] and for drug interventions for chronic migraine [41,82]. For medication trials with episodic migraine, 50% reduction is assumed as an adequate threshold [41]. The observed primary effect in the second data analysis, which assessed migraine days, is a reduction in monthly migraine headache days by 2.40 days, which corresponds to a mean reduction of 44% compared to baseline. 58% of patients can be classified as 30%-responders and 47% as 50%-responders in this data analysis. Thus, the improvement with the use of sinCephalea is clearly above the range that is classified as clinically relevant for non-pharmacological interventions. In addition, the effect lies in a range that is considered as clinically relevant for medications. At the same time, it can be assumed that this clinical efficacy can be achieved without the side effects common with drug therapy. HIT-6 and MIDAS are also used in the evaluation of drug trials. From a mean intra-individual reduction of at least 2.5 points in the HIT-6 score, pharmacological interventions are classified as clinically significant [40,46,83]. This threshold was exceeded in both data analyses with improvements of 3.17 to 4.48 points. A reduction in MIDAS of at least 30% is also assessed as clinically significant [40,49]; in this case, the observed improvements between 27% and 45.5% are again in a range that is considered as clinically relevant for medications. In summary, the assessment tools used in the reported studies suggest a significant clinical effect of sinCephalea adjusted against the threshold values stated in the literature.

The analyses are based on intraindividual pre-post comparisons without the use of parallel control groups. For additional clinical classification of the observed effects, a literature search for studies with comparable, non-pharmacological interventions was conducted. To estimate the potential effect in a control group, eight studies were identified in which migraine patients received standard care in an open control group [58,74,84,85,86,87,88,89]. These control patients were all aware that they belonged to a control group and represent what can be expected as unspecific treatment effects induced by study participation itself or by other context effects. Mean changes in migraine days with corresponding standard deviations and sample sizes were extracted from these publications and meta-analyzed using a random effects model. The pooled mean control group effect of non-drug therapies is a reduction of 1.14 monthly migraine days (random effect model, 95% CI [1.09; 1.25]). The therapeutic effect described in the present data analysis is clearly above the calculated control group effect, even after imputation of missing data. This further supports that the use of sinCephalea can induce clinically relevant effects, and that the observed reduction in migraine days in the present evaluation is above a reduction that can result from non-specific context effects (“placebo effect”) and could withstand a confirmatory test against an open control group.

As a third approach to classify the observed effects, we chose a comparison with the effects in meta-analyses for approved migraine medications. For monoclonal antibodies, the mean reduction in migraine days is reported to be 1.5 days above placebo (95% CI [1.16; 1.85]) or 43% reduction from baseline [90]. For generic medications, effects over placebo of between 0.57 and 1.5 headache days have been reported in meta-analyses, e.g., valproate (−1.5, 95% CI [−2.1; −0.8]), metoprolol (−0.94, 95% CI [−1.4; −0.46]) or fluoxetine (−0.57, 95% CI [−0.97; −0.17]) [71]. This comparison also supports the clinical relevance of the observed effects of sinCephalea.

In conclusion, sinCephalea is a non-pharmacological, digital migraine prophylaxis that is, firstly, used by patients regularly and according to the instructions, and secondly, induces a therapeutic effect that is within the range of pharmacological interventions. To date, no unexpected side effects have been reported from the use of sinCephalea. It is therefore a digital therapeutic that can effectively complement the current standard of care and can be a helpful application in migraine prevention. The implementation of this innovative device can help many migraine patients to improve their quality of life. In addition, sinCephalea can help patients not only to understand their disease, but also to understand their personal metabolic situation. The obtained data provide a first insight into the potential effectiveness of a personalized low-glycemic diet in the prevention of migraine attacks. To be able to prove the actual effectiveness of sinCephalea by comparing the reduction of migraine days with a control group, a confirmatory study including a parallel control group is essential.

## Figures and Tables

**Table 1:** Comparison of disease severity between the four-week baseline phase and the last four-weeks of the intervention phase of the first study (*n* = 49). The disease severity was defined by the number of headache days, the average intensity of headache, Headache Impact Test-6 items (HIT-6), Migraine Disability Score (MIDAS) and quality of life (EQ-5D-5L).

Variable	Time	Mean (SD)	Median	Min	Max	Change [95% CI]	*p*-Value
Headache days (per four weeks)	Baseline	4.26 (3.36)	3.0	1.0	21.0	−2.43 [−3.36; −1.49] −62.55% [−78.32; −46.78]	<0.001
	Intervention	1.83 (3.50)	0.0	0.0	18.0		
Average intensity of headache (0–10)	Baseline	6.29 (1.37)	6.0	4.0	9.0	−0.68 [−1.24; −0.12]	<0.05
	Intervention	5.53 (1.50)	6.0	3.0	8.0		
HIT-6 (Score 36–78)	Baseline	61.80 (4.60)	62.0	44.0	72.0	−4.48 [−6.82; −2.60]	<0.001
	Intervention	57.50 (5.62)	58.0	44.0	67.0		
MIDAS	Baseline	37.6 (58.9)	25.0	0.0	370.0	−11.03 [−21.87; −0.44] −45.5% [−44.66; 5.39]	<0.01
	Intervention	20.3 (20.4)	15.0	0.0	81.0		
EQ-5D-5L (index value)	Baseline	0.891 (0.11)	0.909	0.505	1.000	+4.50 [−0.06; 7.89])	<0.05
	Intervention	0.942 (0.08)	0.999	0.719	1.000		

**Table 2:** Change in migraine headache days, non-migraine headache days, and cumulative duration of migraine headache in hours between the four-week baseline phase and last four weeks of intervention phase in the complete data set analysis (*n* = 62) and in the intention-to-treat analysis (*n* = 71). Data was assessed with the daily headache diary.

Variable	Time	Mean (SD)	Median	Min	Max	Change [95% CI]	*p*-Value
**Complete Data Set (*n* = 62)**							
Migraine headache days	Baseline	7.59 (3.91)	6.15	3.00	16.59	−2.40 [−3.37; −1.42]	<0.001
	Intervention	5.20 (3.61)	5.00	0.00	14.00		
Non-migraine headache days	Baseline	2.54 (3.04)	2.04	0.00	18.31	−1.26 [−1.79; −0.72]	<0.001
	Intervention	1.28 (2.69)	0.00	0.00	18.26		
Cumulative duration of migraine headache	Baseline	61.21 (39.31)	58.99	8.05	182.03	−15.22 [−25.54; −4.90]	0.005
	Intervention	45.99 (39.52)	35.54	0.00	171.21		
**Intention-to-Treat (*n* = 71)**							
Migraine headache days	Baseline	7.78 (3.92)	6.46	3.00	16.59	−2.23 [−3.10; −1.37]	<0.001
	Intervention	5.55 (3.83)	5.00	0.00	15.17		
Non-migraine headache days	Baseline	2.55 (2.98)	2.00	0.00	18.31	−1.14 [−1.62; −0.66]	<0.001
	Intervention	1.40 (2.69)	1.00	0.00	18.26		
Cumulative duration of migraine headache	Baseline	62.79 (38.28)	59.50	8.05	182.03	−14.20 [−23.30; −5.11]	0.001
	Intervention	48.58 (39.45)	36.78	0.00	171.21		

**Table 3:** Change in headache-related and migraine-related impairment in everyday life and quality of life between the four-week baseline phase and the last four weeks of the intervention phase (*n* = 60). Changes were assessed by Headache Impact Test-6 items (HIT-6), Migraine Disability Score (MIDAS) and quality of life (EQ-5D-5L).

Variable	Time	Mean (SD)	Median	Min	Max	Change [95% CI]	*p*-Value
HIT-6 (Score 36–78)	Baseline	64.43 (3.85)	65.00	52.00	74.00	−3.17 [−4.63; −1.70]	<0.001
	Intervention	61.27 (6.34)	62.00	44.00	76.00		
MIDAS	Baseline	53.57 (48.28)	41.50	2.00	279.00	−13.45 [−22.01; −4.89]	0.002
	Intervention	40.12 (45.25)	27.50	0.00	259.00		
EQ-5D-5L (index value)	Baseline	0.87 (0.17)	0.91	0.325	1.00	0.022 [−0.03; 0.08]	0.240
	Intervention	0.89 (0.17)	0.91	0.048	1.00		

## Data Availability

The datasets used and/or analyzed during the current study are available from the corresponding author on reasonable request.

## References

[1] (2018). GBD 2017 Disease and Injury Incidence and Prevalence Collaborators Global, Regional, and National Incidence, Prevalence, and Years Lived with Disability for 354 Diseases and Injuries for 195 Countries and Territories, 1990–2017: A Systematic Analysis for the Global Burden of Disease Study 2017. Lancet.

[2] Lipton R.B., Bigal M.E., Diamond M., Freitag F., Reed M.L., Stewart W.F. (2007). Migraine Prevalence, Disease Burden, and the Need for Preventive Therapy. Neurology.

[3] Stovner L., Hagen K., Jensen R., Katsarava Z., Lipton R., Scher A., Steiner T., Zwart J.-A. (2007). The Global Burden of Headache: A Documentation of Headache Prevalence and Disability Worldwide. Cephalalgia Int. J. Headache.

[4] Yoon M.-S., Katsarava Z., Obermann M., Fritsche G., Oezyurt M., Kaesewinkel K., Katsarova A., Santowski I., Diener H., Moebus S. (2012). Prevalence of Primary Headaches in Germany: Results of the German Headache Consortium Study. J. Headache Pain.

[5] Goadsby P.J., Holland P.R., Martins-Oliveira M., Hoffmann J., Schankin C., Akerman S. (2017). Pathophysiology of Migraine: A Disorder of Sensory Processing. Physiol. Rev..

[6] Statista Umfrage Zur Wahl Des Arztes Bei Kopfschmerzen Unter Studenten in Deutschland 2018. https://de.statista.com/statistik/daten/studie/972749/umfrage/umfrage-zur-wahl-des-arztes-bei-kopfschmerzen-unter-studenten-in-deutschland/.

[7] Diemer W., Burchert H. (2002). Chronische Schmerzen: Kopf-und Rückenschmerzen, Tumorschmerzen.

[8] Statistisches Bundesamt 23631-0001: Krankheitskosten: Deutschland, Jahre, Krankheitsdiagnosen (ICD-10). https://www-genesis.destatis.de/genesis//online?operation=table&code=23631-0001&levelindex=0&levelid=1587706110657.

[9] Bloudek L.M., Stokes M., Buse D.C., Wilcox T.K., Lipton R.B., Goadsby P.J., Varon S.F., Blumenfeld A.M., Katsarava Z., Pascual J. (2012). Cost of Healthcare for Patients with Migraine in Five European Countries: Results from the International Burden of Migraine Study (IBMS). J. Headache Pain.

[10] Bonafede M., Sapra S., Shah N., Tepper S., Cappell K., Desai P. (2018). Direct and Indirect Healthcare Resource Utilization and Costs Among Migraine Patients in the United States. Headache J. Head Face Pain.

[11] Diener H.-C., Holle D., Solbach K., Gaul C. (2016). Medication-Overuse Headache: Risk Factors, Pathophysiology and Management. Nat. Rev. Neurol..

[12] Diener H.-C., Gaul C., Kropp P. (2018). Therapie der Migräneattacke und Prophylaxe der Migräne: Entwicklungsstufe: S1. Nervenheilkunde.

[13] Gobel H., Heinze A., Heinze-Kuhn K., Gobel C.H.H. (2020). Modern migraine therapy-interdisciplinary long-term care. Der. Internist..

[14] Hepp Z., Dodick D.W., Varon S.F., Chia J., Matthew N., Gillard P., Hansen R.N., Devine E.B. (2017). Persistence and Switching Patterns of Oral Migraine Prophylactic Medications among Patients with Chronic Migraine: A Retrospective Claims Analysis. Cephalalgia.

[15] Woolley J.M., Bonafede M.M., Maiese B.A., Lenz R.A. (2017). Migraine Prophylaxis and Acute Treatment Patterns Among Commercially Insured Patients in the United States. Headache.

[16] Diener H.-C., Nägel S., Leitlinie E.D., Migräneattacke T.D., May P.A. (2020). Prophylaxe Episodischer Und Chronischer Migräne Mit CGRP(Rezeptor)-Antikörpern. InFo Neurol. Psychiatr..

[17] Sacco S., Bendtsen L., Ashina M., Reuter U., Terwindt G., Mitsikostas D.-D., Martelletti P. (2019). European Headache Federation Guideline on the Use of Monoclonal Antibodies Acting on the Calcitonin Gene Related Peptide or Its Receptor for Migraine Prevention. J. Headache Pain.

[18] Tiseo C., Ornello R., Pistoia F., Sacco S. (2019). How to Integrate Monoclonal Antibodies Targeting the Calcitonin Gene-Related Peptide or Its Receptor in Daily Clinical Practice. J. Headache Pain.

[19] Siva Z.O., Uluduz D., Keskin F.E., Erenler F., Balcı H., Uygunoğlu U., Saip S., Göksan B., Siva A. (2018). Determinants of Glucose Metabolism and the Role of NPY in the Progression of Insulin Resistance in Chronic Migraine. Cephalalgia Int. J. Headache.

[20] Yilmaz N., Aydin O., Yegin A., Tiltak A., Eren E., Aykal G. (2011). Impaired Oxidative Balance and Association of Blood Glucose, Insulin and HOMA-IR Index in Migraine. Biochem. Med..

[21] Bernecker C., Ragginer C., Fauler G., Horejsi R., Möller R., Zelzer S., Lechner A., Wallner-Blazek M., Weiss S., Fazekas F. (2011). Oxidative Stress Is Associated with Migraine and Migraine-Related Metabolic Risk in Females. Eur. J. Neurol..

[22] Gruber H.-J., Bernecker C., Pailer S., Fauler G., Horejsi R., Möller R., Lechner A., Fazekas F., Truschnig-Wilders M. (2010). Hyperinsulinaemia in Migraineurs Is Associated with Nitric Oxide Stress. Cephalalgia Int. J. Headache.

[23] Vollesen A.L.H., Guo S., Ashina M. (2017). PACAP38 Dose-Response Pilot Study in Migraine Patients. Cephalalgia Int. J. Headache.

[24] Ghanizada H., Al-Karagholi M.A.-M., Arngrim N., Olesen J., Ashina M. (2020). PACAP27 Induces Migraine-like Attacks in Migraine Patients. Cephalalgia Int. J. Headache.

[25] Bongiovanni D., Benedetto C., Corvisieri S., Del Favero C., Orlandi F., Allais G., Sinigaglia S., Fadda M. (2021). Effectiveness of Ketogenic Diet in Treatment of Patients with Refractory Chronic Migraine. Neurol. Sci..

[26] Evcili G., Utku U., Öğün M.N., Özdemir G. (2018). Early and Long Period Follow-up Results of Low Glycemic Index Diet for Migraine Prophylaxis. J. Turk. Soc. Algol..

[27] Schröder T., Kühn G., Kordowski A., Jahromi S.R., Gendolla A., Evers S., Gaul C., Thaçi D., König I.R., Sina C. (2022). A Digital Health Application Allowing a Personalized Low-Glycemic Nutrition for the Prophylaxis of Migraine: Proof-of-Concept Data from a Retrospective Cohort Study. J. Clin. Med..

[28] Berry S.E., Valdes A.M., Drew D.A., Asnicar F., Mazidi M., Wolf J., Capdevila J., Hadjigeorgiou G., Davies R., Al Khatib H. (2020). Human Postprandial Responses to Food and Potential for Precision Nutrition. Nat. Med..

[29] Korem T., Zeevi D., Zmora N., Weissbrod O., Bar N., Lotan-Pompan M., Avnit-Sagi T., Kosower N., Malka G., Rein M. (2017). Bread Affects Clinical Parameters and Induces Gut Microbiome-Associated Personal Glycemic Responses. Cell Metab..

[30] Mendes-Soares H., Raveh-Sadka T., Azulay S., Edens K., Ben-Shlomo Y., Cohen Y., Ofek T., Bachrach D., Stevens J., Colibaseanu D. (2019). Assessment of a Personalized Approach to Predicting Postprandial Glycemic Responses to Food Among Individuals Without Diabetes. JAMA Netw. Open.

[31] Zeevi D., Korem T., Zmora N., Israeli D., Rothschild D., Weinberger A., Ben-Yacov O., Lador D., Avnit-Sagi T., Lotan-Pompan M. (2015). Personalized Nutrition by Prediction of Glycemic Responses. Cell.

[32] (2016). on behalf of the Food4Me Study Effect of an Internet-Based, Personalized Nutrition Randomized Trial on Dietary Changes Associated with the Mediterranean Diet: The Food4Me Study. Am. J. Clin. Nutr..

[33] Deutsche Gesellschaft für Ernährung DGE-Vollwertige Ernährung. https://www.dge.de/ernaehrungspraxis/vollwertige-ernaehrung/.

[34] Hindiyeh N.A., Zhang N., Farrar M., Banerjee P., Lombard L., Aurora S.K. (2020). The Role of Diet and Nutrition in Migraine Triggers and Treatment: A Systematic Literature Review. Headache J. Head Face Pain.

[35] Gazerani P. (2020). Migraine and Diet. Nutrients.

[36] Alm-Roijer C., Stagmo M., Udén G., Erhardt L. (2004). Better Knowledge Improves Adherence to Lifestyle Changes and Medication in Patients with Coronary Heart Disease. Eur. J. Cardiovasc. Nurs..

[37] Hannay D.R. (1981). New Directions in Patient Compliance (Book). Sociol. Health Illn..

[38] Chodankar D. (2021). Introduction to Real-World Evidence Studies. Perspect. Clin. Res..

[39] U.S. Food and Drug Administration Real-World Evidence. https://www.fda.gov/science-research/science-and-research-special-topics/real-world-evidence.

[40] Diener H.C., Ashina M., Durand-Zaleski I., Kurth T., Lantéri-Minet M., Lipton R.B., Ollendorf D.A., Pozo-Rosich P., Tassorelli C., Terwindt G. (2021). Health Technology Assessment for the Acute and Preventive Treatment of Migraine: A Position Statement of the International Headache Society. Cephalalgia.

[41] Diener H.-C., Tassorelli C., Dodick D.W., Silberstein S.D., Lipton R.B., Ashina M., Becker W.J., Ferrari M.D., Goadsby P.J., Pozo-Rosich P. (2020). Guidelines of the International Headache Society for Controlled Trials of Preventive Treatment of Migraine Attacks in Episodic Migraine in Adults. Cephalalgia.

[42] International Headache Society The International Classification of Headache Disorders.

[43] Coeytaux R.R., Kaufman J.S., Chao R., Mann J.D., Devellis R.F. (2006). Four Methods of Estimating the Minimal Important Difference Score Were Compared to Establish a Clinically Significant Change in Headache Impact Test. J. Clin. Epidemiol..

[44] QualityMetric Inc HIT-6TM-Headache Impact TestTM. https://eprovide.mapi-trust.org/instruments/headache-impact-test.

[45] Bjorner J.B., Kosinski M., Ware J.E. (2003). Using Item Response Theory to Calibrate the Headache Impact Test (HIT) to the Metric of Traditional Headache Scales. Qual. Life Res..

[46] Kosinski M., Bayliss M.S., Bjorner J.B., Ware J.E., Garber W.H., Batenhorst A., Cady R., Dahlöf C.G.H., Dowson A., Tepper S. (2003). A Six-Item Short-Form Survey for Measuring Headache Impact: The HIT-6. Qual. Life Res..

[47] Caremark Inc Migraine Disability Assessment (MIDAS) Questionnaire. https://eprovide.mapi-trust.org/instruments/migraine-disability-assessment.

[48] Stewart W.F., Lipton R.B., Whyte J., Dowson A., Kolodner K., Liberman J.N., Sawyer J. (1999). An International Study to Assess Reliability of the Migraine Disability Assessment (MIDAS) Score. Neurology.

[49] Stewart W.F., Lipton R.B., Kolodner K.B., Sawyer J., Lee C., Liberman J.N. (2000). Validity of the Migraine Disability Assessment (MIDAS) Score in Comparison to a Diary-Based Measure in a Population Sample of Migraine Sufferers. Pain.

[50] EuroQol Research Foundation EuroQoL-5 Dimension Questionnaire 2021. https://euroqol.org/eq-5d-instruments/eq-5d-5l-about/.

[51] Stafford M.R., Hareendran A., Ng-Mak D.S., Insinga R.P., Xu R., Stull D.E. (2012). EQ-5D^TM^-Derived Utility Values for Different Levels of Migraine Severity from a UK Sample of Migraineurs. Health Qual. Life Outcomes.

[52] Xu R., Insinga R.P., Golden W., Hu X.H. (2011). EuroQol (EQ-5D) Health Utility Scores for Patients with Migraine. Qual. Life Res..

[53] Ludwig K., Graf von der Schulenburg J.-M., Greiner W. (2018). German Value Set for the EQ-5D-5L. Pharmacoeconomics.

[54] (2020). National Headache Foundation Diet for People with Headache Disorders. https://headaches.org/headache-diet-2020/.

[55] (2016). American Migraine Foundation Migraine and Diet. https://americanmigrainefoundation.org/resource-library/migraine-and-diet/.

[56] (2017). American Migraine Foundation Planning Your Diet Around Your Migraine. https://americanmigrainefoundation.org/resource-library/planning-migraine-diet/.

[57] Deutsche Migräne- und Kopfschmerzgesellschaft Ernährung Und Migräne. https://www.dmkg.de/files/dmkg.de/patienten/Download/ernaehrung.pdf.

[58] Bunner A.E., Agarwal U., Gonzales J.F., Valente F., Barnard N.D. (2014). Nutrition Intervention for Migraine: A Randomized Crossover Trial. J. Headache Pain.

[59] Di Lorenzo C., Pinto A., Ienca R., Coppola G., Sirianni G., Di Lorenzo G., Parisi V., Serrao M., Spagnoli A., Vestri A. (2019). A Randomized Double-Blind, Cross-Over Trial of Very Low-Calorie Diet in Overweight Migraine Patients: A Possible Role for Ketones?. Nutrients.

[60] Ferrara L.A., Pacioni D., Di Fronzo V., Russo B.F., Speranza E., Carlino V., Gargiulo F., Ferrara F. (2015). Low-Lipid Diet Reduces Frequency and Severity of Acute Migraine Attacks. Nutr. Metab. Cardiovasc. Dis..

[61] Lorenzo C.D., Coppola G., Sirianni G., Lorenzo G.D., Bracaglia M., Lenola D.D., Siracusano A., Rossi P., Pierelli F. (2015). Migraine Improvement during Short Lasting Ketogenesis: A Proof-of-Concept Study. Eur. J. Neurol..

[62] Mirzababaei A., Khorsha F., Togha M., Yekaninejad M.S., Okhovat A.A., Mirzaei K. (2020). Associations between Adherence to Dietary Approaches to Stop Hypertension (DASH) Diet and Migraine Headache Severity and Duration among Women. Nutr. Neurosci..

[63] Papetti L., Moavero R., Ferilli M.A.N., Sforza G., Tarantino S., Ursitti F., Ruscitto C., Vigevano F., Valeriani M. (2021). Truths and Myths in Pediatric Migraine and Nutrition. Nutrients.

[64] Razeghi Jahromi S., Togha M., Jafari E., Mohammadianinejad S.E., Haghighi S., Ansari H. (2022). Chapter Fourteen-Role of Diet, Food, and Nutrition in Prevention and Treatment of Headache. Headache and Migraine in Practice.

[65] Razeghi Jahromi S., Ghorbani Z., Martelletti P., Lampl C., Togha M., On behalf of the School of Advanced Studies of the European Headache Federation (EHF-SAS) (2019). Association of Diet and Headache. J. Headache Pain.

[66] Ziegeler C., Brauns G., Jürgens T.P., May A. (2019). Shortcomings and Missed Potentials in the Management of Migraine Patients-Experiences from a Specialized Tertiary Care Center. J. Headache Pain.

[67] Goadsby P.J., Sprenger T. (2010). Current Practice and Future Directions in the Prevention and Acute Management of Migraine. Lancet Neurol..

[68] Lenaerts M.E., Couch J.R. (2007). Medication Overuse Headache. Minerva Med.

[69] Straube A., Pfaffenrath V., Ladwig K.-H., Meisinger C., Hoffmann W., Fendrich K., Vennemann M., Berger K. (2010). Prevalence of Chronic Migraine and Medication Overuse Headache in Germany—The German DMKG Headache Study. Cephalalgia.

[70] Edwards K.R., Potter D.L., Wu S.-C., Kamin M., Hulihan J. (2003). Topiramate in the Preventive Treatment of Episodic Migraine: A Combined Analysis From Pilot, Double-Blind, Placebo-Controlled Trials. CNS Spectr..

[71] Jackson J.L., Cogbill E., Santana-Davila R., Eldredge C., Collier W., Gradall A., Sehgal N., Kuester J. (2015). A Comparative Effectiveness Meta-Analysis of Drugs for the Prophylaxis of Migraine Headache. PLoS ONE.

[72] Dodick D.W., Ashina M., Brandes J.L., Kudrow D., Lanteri-Minet M., Osipova V., Palmer K., Picard H., Mikol D.D., Lenz R.A. (2018). ARISE: A Phase 3 Randomized Trial of Erenumab for Episodic Migraine. Cephalalgia.

[73] Storey J.R., Calder C.S., Hart D.E., Potter D.L. (2001). Topiramate in Migraine Prevention: A Double-Blind, Placebo-Controlled Study. Headache.

[74] Diener H.-C., Kronfeld K., Boewing G., Lungenhausen M., Maier C., Molsberger A., Tegenthoff M., Trampisch H.-J., Zenz M., Meinert R. (2006). Efficacy of Acupuncture for the Prophylaxis of Migraine: A Multicentre Randomised Controlled Clinical Trial. Lancet Neurol..

[75] Goadsby P.J., Reuter U., Hallström Y., Broessner G., Bonner J.H., Zhang F., Sapra S., Picard H., Mikol D.D., Lenz R.A. (2017). A Controlled Trial of Erenumab for Episodic Migraine. N. Engl. J. Med..

[76] Fava A., Pirritano D., Consoli D., Plastino M., Casalinuovo F., Cristofaro S., Colica C., Ermio C., De Bartolo M., Opipari C. (2014). Chronic Migraine in Women Is Associated with Insulin Resistance: A Cross-Sectional Study. Eur. J. Neurol..

[77] Fagherazzi G., El Fatouhi D., Fournier A., Gusto G., Mancini F.R., Balkau B., Boutron-Ruault M.-C., Kurth T., Bonnet F. (2019). Associations Between Migraine and Type 2 Diabetes in Women: Findings From the E3N Cohort Study. JAMA Neurol.

[78] Yamaguchi A., Chiba T., Morishita T., Nakamura A., Inui T., Yamatani T., Kadowaki S., Chihara K., Fukase M., Fujita T. (1990). Calcitonin Gene-Related Peptide and Induction of Hyperglycemia in Conscious Rats in Vivo. Diabetes.

[79] Durham P.L. (2006). Calcitonin Gene-Related Peptide (CGRP) and Migraine. Headache.

[80] Gross E.C., Lisicki M., Fischer D., Sándor P.S., Schoenen J. (2019). The Metabolic Face of Migraine—From Pathophysiology to Treatment. Nat. Rev. Neurol..

[81] Luedtke K., Basener A., Bedei S., Castien R., Chaibi A., Falla D., Fernández-de-las-Peñas C., Gustafsson M., Hall T., Jull G. (2020). Outcome Measures for Assessing the Effectiveness of Non-Pharmacological Interventions in Frequent Episodic or Chronic Migraine: A Delphi Study. BMJ Open.

[82] Dodick D.W., Turkel C.C., DeGryse R.E., Diener H.-C., Lipton R.B., Aurora S.K., Nolan M.E., Silberstein S.D. (2015). Assessing Clinically Meaningful Treatment Effects in Controlled Trials: Chronic Migraine as an Example. J. Pain.

[83] Smelt A.F.H., Assendelft W.J.J., Terwee C.B., Ferrari M.D., Blom J.W. (2014). What Is a Clinically Relevant Change on the HIT-6 Questionnaire? An Estimation in a Primary-Care Population of Migraine Patients. Cephalalgia.

[84] Darabaneanu S., Overath C.H., Rubin D., Luthje S., Sye W., Niederberger U., Gerber W.-D., Weisser B. (2011). Aerobic Exercise as a Therapy Option for Migraine: A Pilot Study. Int. J. Sports Med..

[85] Hanssen H., Minghetti A., Magon S., Rossmeissl A., Rasenack M., Papadopoulou A., Klenk C., Faude O., Zahner L., Sprenger T. (2018). Effects of Different Endurance Exercise Modalities on Migraine Days and Cerebrovascular Health in Episodic Migraineurs: A Randomized Controlled Trial. Scand. J. Med. Sci. Sports.

[86] Jena S., Witt C.M., Brinkhaus B., Wegscheider K., Willich S.N. (2008). Acupuncture in Patients with Headache. Cephalalgia.

[87] Krøll L.S., Hammarlund C.S., Linde M., Gard G., Jensen R.H. (2018). The Effects of Aerobic Exercise for Persons with Migraine and Co-Existing Tension-Type Headache and Neck Pain. A Randomized, Controlled, Clinical Trial. Cephalalgia.

[88] Linde K., Streng A., Jürgens S., Hoppe A., Brinkhaus B., Witt C., Wagenpfeil S., Pfaffenrath V., Hammes M.G., Weidenhammer W. (2005). Acupuncture for Patients With Migraine: A Randomized Controlled Trial. JAMA.

[89] Vickers A.J., Rees R.W., Zollman C.E., McCarney R., Smith C.M., Ellis N., Fisher P., Van Haselen R. (2004). Acupuncture for Chronic Headache in Primary Care: Large, Pragmatic, Randomised Trial. BMJ.

[90] Forbes R.B., McCarron M., Cardwell C.R. (2020). Efficacy and Contextual (Placebo) Effects of CGRP Antibodies for Migraine: Systematic Review and Meta-Analysis. Headache.

